# Supplementary material for: Effect of Bacillus subtilis on antioxidant enzyme activities in tomato grafting
Source: PeerJ. 2021 Mar 12;9:e10984. doi: 10.7717/peerj.10984 (PMC7958894; doi:10.7717/peerj.10984)
Supplement: Supplemental Information 9 [file peerj-09-10984-s009.pdf]

	PC1	PC2	PC3	PC4	PC5	PC6	PC7
CAT	-0.47434	0.142211	0.330812	-0.00236	0.239513	-0.07657	-0.76296
SOD	-0.39498	0.330215	0.410167	0.104272	-0.00924	-0.52024	0.533953
POD	-0.50329	0.143918	-0.09722	0.316864	0.127097	0.728421	0.263398
PPO	-0.36698	-0.38447	-0.39926	-0.43915	0.54345	-0.19803	0.175213
PAL	0.239494	0.503151	-0.4631	0.435764	0.471019	-0.23651	-0.08565
Total phenols	-0.41621	0.012159	-0.56922	0.123886	-0.63117	-0.25221	-0.15899
Graft success	-0.02058	-0.67	0.122494	0.700451	0.104004	-0.18474	-0.00995

Table S1. Coefficient of the 7 principal components (Eigenvectors)
