# Supplementary material for: Effect of Bacillus subtilis on antioxidant enzyme activities in tomato grafting
Source: PeerJ. 2021 Mar 12;9:e10984. doi: 10.7717/peerj.10984 (PMC7958894; doi:10.7717/peerj.10984)
Supplement: Supplemental Information 10 — Positive values mean inoculated plants had higher activity (or total phenol content). Negative values mean control plants had higher activity (or total phenol content). CAT:Catalase, SOD:Superoxide dismutase, POD:Peroxidase, PPO: Polyphenol oxidase, PAL:Phenylalanine ammonia-lyase. RGCh: “Rio Grande” grafted on “Cherry”, RGBer: “Rio Grande” grafted on eggplant. [file peerj-09-10984-s010.pdf]

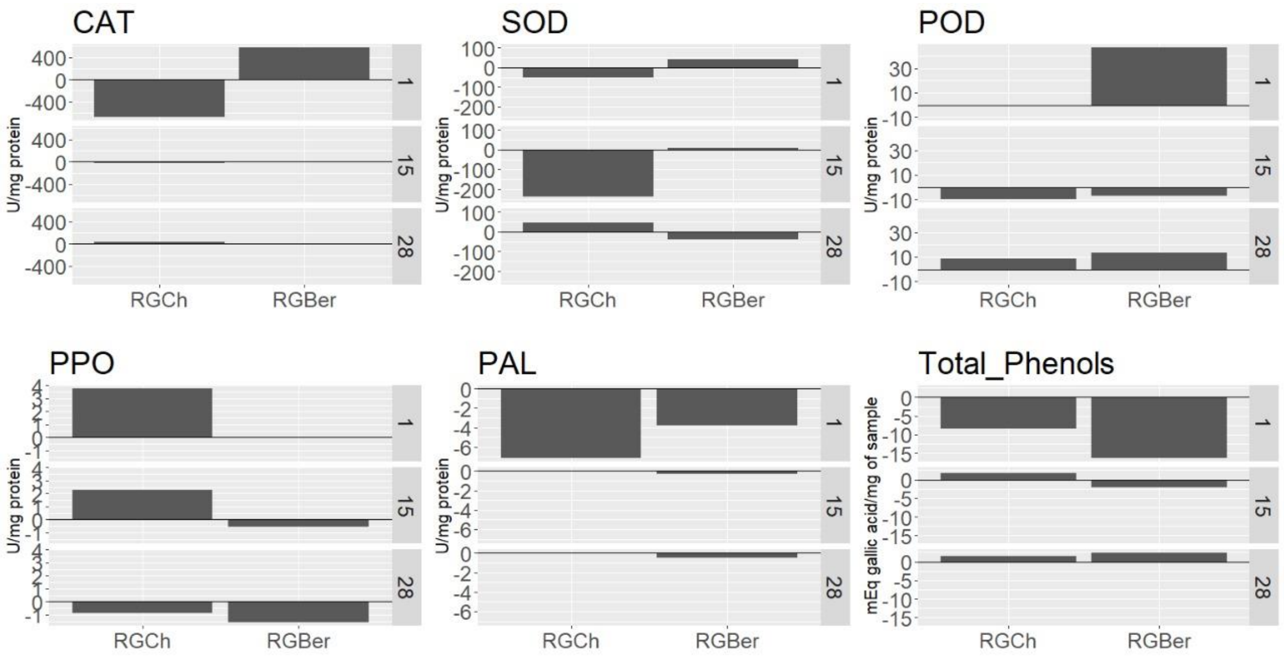

Figure S1. Enzyme activities (CAT, SOD, POD, PPO, PAL) and total phenols given by the difference between inoculated and control plants. Rio Grande" grafted on "Cherry" (RGCh) and "Rio Grande" grafted on eggplant (RGBer)

Positive values mean inoculated plants had higher activity (or total phenol content). Negative values mean control plants had higher activity (or total phenol content). CAT:Catalase, SOD:Superoxide dismutase, POD:Peroxidase, PPO: Polyphenol oxidase, PAL:Phenylalanine ammonia-lyase. RGCh: "Rio Grande" grafted on "Cherry", RGBer: "Rio Grande" grafted on eggplant.
